# Healthy sexuality development in adolescence: proposing a competency-based framework to inform programmes and research

**DOI:** 10.1080/26410397.2021.1996116

**Published:** 2021-12-23

**Authors:** Anna Kågesten, Miranda van Reeuwijk

**Affiliations:** a Assistant Professor, Department of Global Public Health, Karolinska Institutet, Stockholm, Sweden. *Correspondence:* anna.kagesten@ki.se; b Senior Researcher, Rutgers, Utrecht, The Netherlands

**Keywords:** adolescents, sexuality, sexual wellbeing, SRHR, positive approach, competencies, development, framework

## Abstract

Positive aspects of sexuality remain understudied among young people globally, and consensus is lacking on how to conceptualise different aspects of healthy adolescent sexuality development in order to guide programmes, research, and policy. We propose a conceptual framework that draws on theories and literature related to positive youth development, empowerment, human rights, gender, social-ecological and life-course perspectives. The framework highlights six key competencies for healthy adolescent sexuality development: (1) sexual literacy, (2) gender-equal attitudes, (3) respect for human rights and understanding consent, (4) critical reflection skills, (5) coping skills, and (6) interpersonal skills. These competencies have the potential to strengthen or impede adolescents’ sense of sexual wellbeing in relation to both themselves (e.g. body image, self-efficacy) and others (e.g. mutually respectful relationships). Whether adolescents are able to translate competencies into desired actions and achieve a sense of sexual wellbeing depends on the resources available to them, their agency, and on the influence of social-ecological opportunity structures. The framework can provide concrete direction for sexual and reproductive health practitioners and researchers by providing a platform for recognising and operationalising indicators of healthy sexuality development, and serve as a Theory of Change for programmes aiming to improve adolescent sexual and reproductive health and wellbeing. Our assets-based, life-course approach can also be used to make the case to donors and policymakers for why early investments and positive approaches to adolescent sexuality are needed to achieve sexual wellbeing over time.

## Résumé

Des aspects positifs de la sexualité demeurent sous-étudiés chez les jeunes dans le monde, et un consensus fait défaut sur la manière de conceptualiser différents aspects d'un développement sain de la sexualité chez les adolescents afin de guider les programmes, la recherche et les politiques. Nous proposons un nouveau cadre conceptuel qui s'inspire de théories et de publications relatives au développement positif des jeunes, à leur autonomisation, aux droits de l'homme, au genre, aux perspectives socio-écologiques et du parcours de vie. Ce cadre met en évidence six compétences clés pour un développement sain de la sexualité chez les adolescents : 1) les connaissances essentielles sur la sexualité ; 2) des attitudes basées sur l'égalité des sexes ; 3) le respect des droits de l'homme et la compréhension de la notion de consentement ; 4) les aptitudes de réflexion critique ; 5) les capacités d'adaptation ; et 6) les capacités interpersonnelles. Ces compétences ont le potentiel de renforcer ou d'entraver le sentiment de bien-être sexuel chez les adolescents en rapport aussi bien avec eux-mêmes (par exemple l'image de leur corps, l'auto-efficacité) qu'avec les autres (par exemple des relations dans le respect mutuel). La capacité des adolescents de traduire les compétences en actions souhaitées et de parvenir à un sentiment de bien-être sexuel dépend des ressources dont ils disposent, de leur pouvoir et de l'influence des structures d'opportunités socio-écologiques. Le cadre peut donner une orientation concrète aux praticiens et aux chercheurs en santé sexuelle et reproductive en offrant une plateforme pour reconnaître et opérationnaliser des indicateurs d'un développement sain de la sexualité. Il sert également de théorie du changement pour les programmes souhaitant améliorer la santé et le bien-être sexuels et reproductifs chez les adolescents. Notre approche, fondée sur les atouts et englobant le parcours de vie, peut aussi être utilisée pour montrer aux donateurs et aux décideurs les raisons pour lesquelles des investissements précoces et des approches positives de la sexualité des adolescents sont nécessaires pour parvenir au bien-être sexuel au fil du temps.

## Resumen

Los aspectos positivos de la sexualidad continúan siendo poco estudiados entre las personas jóvenes mundialmente y se carece de consenso en cuanto a cómo conceptualizar los diferentes aspectos del desarrollo saludable de la sexualidad adolescente a fin de guiar los programas, las investigaciones y las políticas. Proponemos un nuevo marco conceptual basado en teorías y literatura relacionadas con el desarrollo positivo, empoderamiento, derechos humanos, género y perspectivas socioecológicas y del ciclo de vida de la juventud. El marco destaca seis competencias clave para el desarrollo saludable de la sexualidad adolescente: 1) alfabetización sexual, 2) actitudes de igualdad de género, 3) respeto por los derechos humanos y comprensión acerca del consentimiento, 4) habilidades de reflexión crítica, 5) habilidades de afrontamiento y 6) habilidades interpersonales. Estas competencias tienen el potencial de fortalecer o impedir un sentido de bienestar sexual de las personas adolescentes con relación a sí mismas (ej., imagen corporal, autoeficacia) y a otras personas (ej., relaciones mutuamente respetuosas). La capacidad de la adolescencia para traducir sus competencias a acciones deseadas y lograr un sentido de bienestar sexual depende de los recursos a su disposición, de su agencia y de la influencia de estructuras de oportunidades socioecológicas. El marco puede ofrecer una dirección concreta para profesionales de salud sexual y reproductiva e investigadores, al proporcionar una plataforma para reconocer y operacionalizar los indicadores del desarrollo saludable de la sexualidad, y servir como Teoría de Cambio para los programas destinados a mejorar la salud sexual y reproductiva y el bienestar sexual de la adolescencia. Nuestro enfoque basado en recursos y en el ciclo de vida también puede utilizarse para presentar ante donantes y formuladores de políticas los argumentos a favor de inversiones tempranas y enfoques positivos en la sexualidad adolescente necesarios para lograr bienestar sexual a lo largo del tiempo.

## Introduction

Positive aspects of sexuality remain understudied among young people globally,^1–7^ especially in low- and middle-income countries,^5,8^ and consensus is lacking on how to conceptualise different aspects of adolescent sexual wellbeing.^5,9^ In this review article, we synthesise the literature and propose a conceptual framework for healthy adolescent sexuality development with the goal of offering guidance to programmes and research aiming to improve sexual and reproductive health and rights (SRHR). The framework is based on an extensive narrative review of the literature to identify key components, followed by input from over 20 global experts in the field of adolescent SRHR during workshops, conferences and webinars. We begin with a background on adolescent sexuality development and wellbeing based on existing definitions and conceptualisations. We then move on to explain the different components of the framework and discuss its potential implications for medical and public health professionals. We end with reflection on the next steps in terms of refining and validating the framework.

## Background

### Moving away from a risk-based approach to adolescent sexuality

Historically, most research studies and programmes have viewed adolescent sexuality as a problem behaviour or “sickness best prevented” that is both immoral and socially unaccepted – if outside of marriage – and brings both physical (e.g. HIV, unintended pregnancies) and psychological consequences (e.g. emotional trauma, depressive symptoms, academic problems).^2,3,7^ Indeed, Tolman and McClelland^7^ found that 80% of studies on adolescent sexuality in the first decade of the twenty-first century focused on the prevention of negative outcomes. While the latter is not strange from a public health perspective (certainly, it is a lot easier to justify studying a sensitive topic because of its potential risks, as opposed to focusing on positive aspects which may be both taboo and difficult to measure),^2^ an exclusive risk focus is problematic in a number of ways.

First, it assumes that adolescent sexuality and related behaviours are *a priori* problematic, while in fact they are part of a normative developmental process. From a life-course perspective, sexuality development begins at (or even before) birth, and includes various behavioural, relational and normative transitions throughout the life course – with adolescence being one such key transition.^10,11^ During puberty, sexual development is accelerated via growth of genitals and hormonal changes, manifesting both physically and emotionally. With growing moral and cognitive development, adolescents increasingly develop abstract reasoning and problem-solving skills, and reflect on their own personal qualities as well as their place in the world – and how they fit in relation to others. Young people gradually develop their sexual orientation, and start to form romantic and sexual relationships and to experiment with different practices.^10^ Today, sexual initiation during adolescence (within or outside of marriage) is normative in most cultural settings, and the emergence of new contraceptive methods and abortion has further separated sex from pregnancy and childbearing.^3^ Second, risk and danger-oriented interventions including abstinence-only programmes have been shown not to be effective in preventing sexual risk; and to clash with adolescent’s needs, realities and human rights to explore their sexuality, and to seek and receive SRHR information and services.^6,12^ And finally, because sexual activity is almost always conceptualised in terms of penile-vaginal intercourse, and rarely in terms of relationships, pleasure, feelings and other sexual practices, programmes tend to focus on older, heterosexual youth – thereby overlooking what is needed to promote healthy sexuality development among a diversity of adolescents, including sexual minorities and younger age groups.^3^

A growing chorus of voices from scholars and stakeholders are therefore calling for positive or healthy approaches to adolescent SRHR.^1–7^ Such a perspective does not mean that all young people are (or should be) having sex, or that all intimate relations are healthy. It merely implies that adolescent sexuality involves more than (just) avoiding risks and unwanted consequences.^1,3,4^ For example, for some young people sexual abstinence may be the healthiest potential choice at one point in time, and for some it may involve other ways of exploring sexuality than intercourse *per se*. As such, empowering young people with different skills (e.g., social, emotional, cognitive) that allow them to navigate information and relationships is an essential part of development for *all* adolescents, irrespective of whether or not they are sexually active.^1^

### A rights-based approach to adolescent sexuality

Positive views on adolescent sexuality are closely connected to a *rights-based approach*, which holds that all adolescents have the right to sexual and reproductive health information, self-determination and civil engagement. Rights-based approaches move beyond disease prevention to emphasise healthy sexuality development, empowerment and individuals’ engagement with their surrounding communities. They do this by focusing on issues that are central to the day-to-day lives of adolescents, such as the navigation of gender norms, identity formation, sexual orientation, relationship power, and broader social and cultural messages.^13,14^ Rights-based approaches also involve participatory teaching techniques aiming to engage young people in critical thinking.^15^ There is growing evidence that a rights-based approach can have positive effects on sexual health. For example, a 2015 review found that the most effective sexuality education programmes are those that integrate content on gender and power.^16,17^ In addition, evidence from the US shows that a comprehensive sexuality education using a rights-based curriculum also has the potential to affect psychosocial outcomes such as greater knowledge about sexual health, more positive attitudes towards sexual relationship rights, more self-efficacy to mitigate sexual risks in relationships, and greater communication about sexuality and relationships with parents.^13^

### Adolescent sexuality as a developmental process

Adolescent sexuality development does not occur in isolation, but interacts with other developmental facets (e.g. the formation of morality, values, identity), and is shaped by the complex interplay between individuals and their social-ecological environment.^2,3,7^ Such a perspective is rooted in Developmental Systems Theory and Positive Youth Development (PYD), which suggests young people should be seen and nurtured as resources rather than being problematised.^18,19^ The premise of PYD is that in order to promote and sustain healthy outcomes in adolescence, programmes should focus on strengthening five central domains of developmental assets, sometimes called the “five Cs” of PYD – *Competence* (e.g. social and academic skills), *Confidence* (e.g. sense of positive self-worth), *Character* (e.g. understanding of norms and mortality), *Connection* (e.g. positive bonds with others) and *Caring* (e.g. sense of sympathy and empathy). These domains are interactive, meaning that adolescents “thrive” when they experience healthy development within each of the five Cs.^19^ Evidence shows that building on adolescents’ developmental assets in line with PYD can have both immediate and long-term positive effects on their health and wellbeing.^20^ For example, Blum et al.^10^ propose four central outcomes of healthy development during early adolescence, including: life and decision-making skills, self-efficacy, emotional and physical safety, and engagement in learning; each of which have been linked with sexual and reproductive health (e.g. contraceptive use, lower risk of STIs) later in adolescence, and are thus closely connected to healthy sexuality development. Further, a 2016 review found that positive aspects of sexuality are linked not only with sexual health, but with physical and mental health as well overall wellbeing.^21^

As noted by Halpern,^2^ “a fine line may divide exploratory sexual activity that ultimately contributes to positive sexual identity and competence, and sexual activity that significantly increases risk of harm”. From a PYD perspective, the role of SRHR interventions is to help young people navigate that “fine line” which may divide potentially positive sexual experiences from those that are harmful.^2^ In order to do so, programmes and research need to include meaningful efforts to understand and strengthen young people’s developmental assets, ultimately affecting their (sexual) wellbeing.

Nonetheless, consensus is lacking around how to define and measure broader, affirmative aspects of adolescent sexuality development,^5,9^ including during the earliest years (ages 10–14) which is a critical period for shaping attitudes and behaviours,^10^ in order to inform research and practice. Below we take a closer look at existing definitions and frameworks that have attempted to conceptualise healthy sexuality and related constructs.

### Defining healthy sexuality and sexual wellbeing: a brief snapshot

Applying a positive lens towards sexuality is in line with several international definitions. For example, the World Health Organization (WHO) emphasises that *sexuality* is a “ … central aspect of being human” that “is experienced and expressed in thoughts, fantasies, desires, beliefs, attitudes, values, behaviours, practices, roles and relationships”. The WHO further defines *sexual health* as a “state of physical, emotional, mental and social *wellbeing in relation to sexuality*”, including the possibility of experiencing “pleasurable and safe sexual experiences”, and emphasising individuals’ sexual rights, such as that to equality and non-discrimination, privacy, information and education about sexuality, freedom of opinion and expression, to choose if and with whom to be sexually active, and to make decisions related to marriage, pregnancy and childbirth.^22^

Such a positive, rights-based view on sexuality is further underscored in the 2018 Guttmacher-Lancet Commission integrated definition of *sexual and reproductive health and rights* (SRHR). This definition draws on already-existing international agreements and documents, emphasising how sexual health is intrinsically linked with sexual rights, reproductive health and reproductive rights. It explicitly mentions that a “positive approach to sexuality and reproduction should recognize the part played by pleasurable sexual relationships, trust and communication in the promotion of self-esteem and overall wellbeing”.^23^ Similarly, over two decades ago the US National Commission on Adolescent Health defined *sexual health* as the “ability to develop and maintain meaningful interpersonal relationships; appreciate one’s own body; interact with both genders in respectful ways; and express affection, love and intimacy in ways consistent with one’s own values”.^24^

In their recent (2021) framework for sexual wellbeing in mid and later life, Mitchell and colleagues^25^ further define *sexual wellbeing* as distinct from (rather than adjunctive to) sexual health. They do so by placing sexual wellbeing and sexual health alongside sexual pleasure and sexual justice as four intersecting pillars that underlie public health studies and programmes. Sexual wellbeing is conceptualised as spanning seven domains: “sexual safety and security, sexual respect, sexual self-esteem, resilience in relation to past sexual experience, forgiveness of past sexual events, self-determination in one’s sex life, and comfort with one’s sexuality”^25^ – all of which applies to people regardless of whether they are sexually active or in relationships. The authors points to the need to monitor sexual wellbeing outcomes as important public health indicators in their own right, e.g. as markers of health equity, so as to recognise the role of context, transgenerational trauma, and needs of marginalised populations.

In addition, a few scholars have focused specifically on conceptualising adolescent sexuality from an affirmative perspective, using overlapping terminologies such as “healthy”, “normative” or “positive” as well as “sexuality”, “sexuality development”, “sexual wellbeing” or “sexual health”.

Hensel and Fortenberry^26^ conceptualise *adolescent sexual health* as spanning four domains: “physical” (e.g. sexual satisfaction), “mental” (e.g. condom efficacy), “social” (e.g. sexual communication) and “emotional” (e.g. relationship quality). Using data from a longitudinal cohort of US adolescent girls, the authors found that girls’ sexual health can be measured as a multi-dimensional construct spanning these domains, which in turn is linked with different sexual prevention behaviours such as condom use and sexual abstinence – making sexual health an “important construct for promoting positive sexual development” regardless of adolescents’ experiences.^26^

Harden^3^ define *adolescent sexual wellbeing* as spanning four key dimensions: “sexual self-esteem” (e.g., perceiving oneself as sexually desirable); “sexual self-efficacy” (e.g., thinking that one is able to express those desires); to have “feelings of arousal, satisfaction, and pleasure as well as recognizing that one is entitled to these feelings”; and “freedom from pain, anxiety, and negative effects regarding sexuality” – irrespective of whether or not a young person has engaged in any sexual behaviours. Not engaging in sex (abstinence) could thus reflect a high sense of wellbeing in the form of an active choice, or low levels of wellbeing (for example via low sexual self-esteem). Harden argues that these dimensions of sexual wellbeing can be thought of as a type of “sexual subjectivity”, defined by Shalet^27^ as:

*“the capacity to be aware of one’s sexual feelings, to enjoy sexual desire and pleasure, to conceive of oneself as the subject [rather than the object] of one’s sexual activities, and to experience a certain amount of control in sexual relationships.”*^3^

Similarly, Anderson^21^ defines *positive aspects of sexuality* as including sexual self-efficacy, sexual self-esteem, sexual pleasure and sexual satisfaction, pointing to sexual satisfaction as an overarching concept encompassing both pleasure and self-esteem. She conceptualises sexual self-efficacy as overlapping with the concept of sexual subjectivity, or the “capability to act as a subject in one’s sexual life”, for example by asserting preferences and boundaries, whereas sexual self-esteem refers to the “confidence and comfort that one feels with their own sexuality” including to communicate with others.

McKee and colleagues^28^ further describe *healthy adolescent sexual development* as having a basic understanding (e.g. of the body) as well as positive attitudes and skills to support sexuality development. They emphasise agency (being in control of one’s own body and decisions) and resilience (learning from bad experiences) as key dimensions, and that a life-course perspective is central where children are “naturally curious about their bodies and sexualities”.^28^ While the authors highlight the complex, multidimensional nature of sexuality development, the description of each of the 15 domains is not very detailed, and it is not clear exactly how they interact and relate to each other.

A more elaborated framework for *positive adolescent sexuality development* is proposed by Arbeit,^1^ focused specifically on skills that allow young people to engage positively with their own sexuality, interact with others, and contribute meaningfully to society focus, including: “sexual selfhood”, “sexual negotiation with others”, and “sexual empowerment”, which in turn are linked via personal intimacy, agency and social advocacy. Specifically, Arbeit argues that an individual’s sexual selfhood may shape their personal agency, which in turn determines their ability to negotiate with others (e.g. by obtaining or giving consent). Negotiations in relationships may in turn affect intimacy, which can lead to sexual empowerment of a young person to advocate for personal needs, thereby affecting their sexual selfhood. How and when adolescents develop these skills vary and is shaped by multiple interacting biological, cultural and social influences such as gender, race, class and contexts.^1^

#### Rationale for proposing a new framework

Based on our review of the above definitions and frameworks, we note that most existing models tend to be academic/theoretical rather than application oriented in their nature, thereby limiting the application to programme development and evaluation. And even for those that are more focused on skills (such as Arbeit’s model)^1^ or that more explicitly operationalise sexual wellbeing outcomes (such as Mitchell and colleagues),^25^ they seem to be (more) applicable to (older) age groups who are already in relationships and/or sexually active, and less so to the earliest years of adolescence. Comprehensive sexuality education, for example, aims not only to prevent traditional public health outcomes but to improve “soft outcomes” such as gender equal norms, consent communication and perceived self-efficacy in relation to *different* age groups (from early childhood to late adolescence).^29^ Design and evaluations of such programmes would thus benefit from a framework that more concretely operationalises healthy sexuality development over the course of adolescence and places this in relation to wellbeing outcomes. We also note that all frameworks were primarily developed by Western researchers; and that research on healthy adolescent sexuality in other parts of the world, including low- and middle-income countries, is largely missing.

Nonetheless, what becomes clear across the frameworks and definitions is that they all emphasise healthy sexuality development as encompassing knowledge, skills and attitudes, which in turn affect individuals’ sense of sexual wellbeing in relation to *themselves* and *others*. Indeed, a recent review found that operational definitions of sexual wellbeing span individual, interpersonal and sociocultural dimensions, and highlighted the need to apply a capability lens that focuses on what young people are “able to be and do” rather than what they “have and do”.^9^ In essence, this means that healthy sexuality development and wellbeing is intrinsically linked with *sexual empowerment*, where agency and competencies or resources play a key role in determining adolescents’ ability to navigate their surrounding contexts.

### Empowerment as central to healthy sexuality development: focusing on competencies

Sexual empowerment can be thought of as the processes of expanding young people’s choice and strengthening their voice related to sexuality through the transformation of power relations, whereby they are able to assert their opinions and desires, influence and make purposeful decisions, and challenge existing norms and structures with respect for consent and the rights of others.^30,31^ A key component of empowerment is agency, which refers to the ability to make/influence decisions and assert own interests and opinions.^32^ Agency is closely connected to the resources available to a person – including both those proximate (e.g. social capital) as well as surrounding opportunity structures at a more distal level such as norms, laws and policies, physical, cultural and economic environment.^31,32^

A useful approach to thinking about the role of resources and its interaction with agency is the conceptual framework for gender transformative life skills programming developed by Kwauk and Braga.^33^ Similar to a PYD approach, this framework conceptualises “life skills” as the combination of *knowledge* (“what one knows”, *attitudes* (“what one believes and values”) and *skills* (“what one has”) into a “broader set of competencies” that allow young people to “function, thrive and adapt in their lived realities”.^33^ By focusing on knowledge, attitudes and skills as different but interlinked competencies, the authors aim to broaden the narrow focus on skills (e.g. self-efficacy) and bring attention to how such resources can be used for transformative change. This is where agency and opportunity structures come into play. Whether or not a young person is able to translate competencies into empowered action (towards desires outcomes) depends on their own agency and the agency of others, as well as the degree to which their social context enables change (via opportunity structures).

## A conceptual framework for adolescent sexual wellbeing

**Figure 1.**
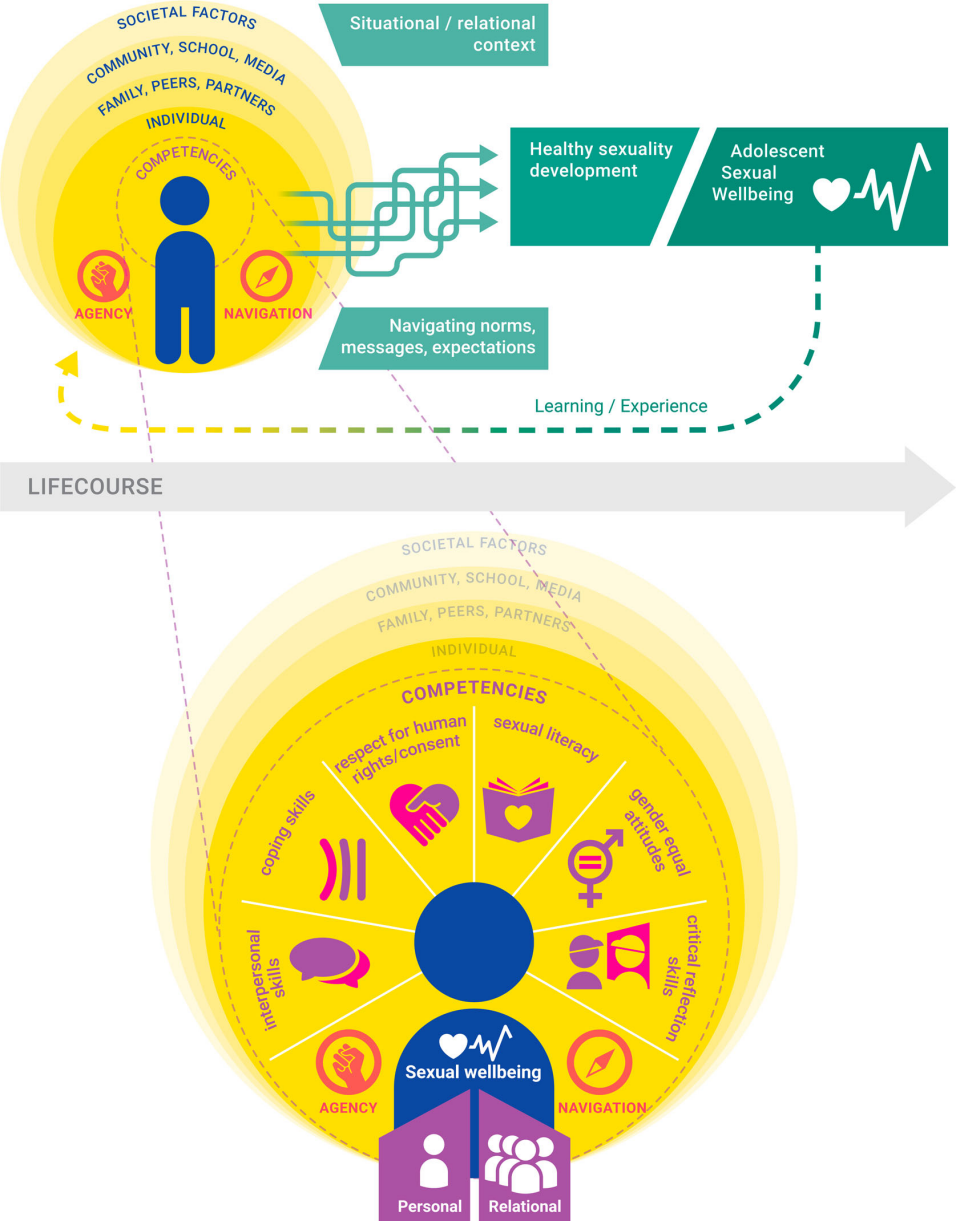
Conceptual framework for healthy adolescent sexuality development and its potential link with sexual wellbeing

Figure 1 presents a conceptual framework for healthy adolescent sexuality development that draws on theories and literature related to PYD, empowerment, human rights, gender, social-ecological and life-course perspectives, and is intended to guide SRHR programmes and research in ages 10–19 years and beyond. The framework is based on an extensive narrative review and synthesis of the definitions described above, where we coded each proposed domain of healthy sexuality (or similar terminologies) to identify key themes and sub-themes. We used these themes to develop an initial draft which was reviewed and discussed during a workshop with experts in the field of adolescent SRHR and sexuality education, hosted by Rutgers in October 2018. Based on the input received, we developed a revised draft which received further input from key partners during global conferences as well as virtual and on-site meetings in 2019.

Drawing on the literature, we differentiate between *healthy sexuality development* as the process through which young people build competencies in the form of knowledge, skills and attitudes that support *sexual wellbeing* in relation to themselves and others. Specifically, we propose six interrelated domains of key competencies that are central to healthy adolescent sexuality development, whether or not sexual activities occur. We view healthy sexuality development as being closely linked with sexual empowerment, and not limited to the “normal” biological developmental phases of adolescence. Whether young people are able to translate competencies into desired actions and achieve a sense of sexual wellbeing across the life-course in turn depends on the personal, social, medical and economic *resources* available to them, on their (sexual) *agency*, and on the influence of social-ecological *opportunity structures*. Strengthening healthy sexuality development thus involves empowering young people to feel comfortable with the normative physical, emotional, cognitive and social (sexual) changes that they are going through, to navigate their surrounding contexts and to form mutually respectful relationships and interactions. For example, it is not only about *having* (normative) feelings of desire, attraction and arousal, but being able to *navigate* and *understand* these feelings.

Below we explain the framework components in more detail.

### Underlying principles

The framework is guided by five key principles:

1. First, *multiple, interlinked social-ecological factors* shape adolescents’ (sexuality) development and wellbeing, including – but not limited to – opportunity structures at individual (e.g. age, sex, socioeconomics, biology, past experiences and trauma, broader attitudes and values); family (e.g. parental connectedness, family structure); peers (e.g. peer norms, number of close friends,); school (e.g. school aspirations, connectedness with teachers); neighbourhood (e.g. safety, cohesion), health systems (e.g. access to youth-friendly health services) and wider societal levels (e.g. laws, policies, social norms, historical events).^10^
2. Second, a *life-course perspective*^11^ is implicit in the framework given that the process of sexuality development is something that occurs from (or even before) birth,^7^ with adolescence being a key transition point of developmental changes.^10,11^
3. Third, it draws on a *human rights-based, gender-transformative* approach to recognise adolescents’ fundamental right to sexual and reproductive health information, services, participation and non-discrimination.^13,14,23^ It recognises gender equality, and particularly equitable gender norms, as central to adolescent development and wellbeing – in line with global evidence that gender transformative programmes can successfully impact health-related knowledge, attitudes and behaviours,^34^ and that healthy adolescent development thus includes the ability to identify and critically examine rigid gender norms.^16^
4. Fourth, it uses a*n empowerment-based* approach^30–32^ and capability lens^9^ to highlight how adolescents’ assets (in the form of competencies) interact with agency and opportunity structures to affect sexual wellbeing. In essence, is about building and nurturing assets in line with the “five C’s” of PYD, including confidence, character and caring.^19^
5. Finally, it emphasises the *reciprocal and complex nature* of sexuality development,^1,3,35^ shown in how the framework components are not linear, but dynamic and interactive, and by pointing to the interconnection between different competencies. Similarly, past experiences inform new ones: for example, if young people are subject to bullying, this will likely affect their attitudes and how they go about forming relationships in the future. For this reason, it is important not to view the different framework components as a checklist that one has to achieve, i.e. failure to achieve certain outcomes does not mean that one is “unhealthy”.

### Key competencies to support healthy sexuality development

Drawing on empowerment theory, the framework highlights six key domains of competencies for healthy sexuality development in the form of *knowledge, skills and attitudes*: (1) sexual literacy, (2) gender equitable attitudes, (3) respect for human rights and understanding of consent, (4) critical reflection skills, (5) coping skills and stress management, and (6) interpersonal relationship skills. These competencies have been introduced briefly elsewhere,^36^ and can be thought of as internal resources to support healthy sexuality development irrespective of whether a young person has engaged in any sexual activities. These competencies move beyond mere knowledge about sexual and reproductive health to span aspects such as coping and communication skills, critical consciousness, and developing personal values that support gender equality and human rights. Many of these competencies are similar to those described in Arbeit’s^1^ skills-based model for adolescent sexuality development and can act as boosters as well as barriers for sexual wellbeing from a personal as well as relational perspective.^1,37^ They are also very much in line with the knowledge, attitudinal and skills-based learning objectives laid out in the International Technical Guidelines for Comprehensive Sexuality Education.^29^

Table 1 provides an overview of definitions, examples of potential contributions to sexual wellbeing as well as operationalisations for each competency.

**Table 1.** Examples of possible contribution to sexual wellbeing as described in the cited references (adapted from Mitchell et al. [25])

Domain	Definition	Examples of possible contribution to sexual wellbeing	Potential operationalisation (measures or indicators)
Sexual literacy	Basic understanding of the human body, relationships and SRHR that is developmentally and age-appropriate^7,23,28,29^	Sexual and reproductive health information associated with sub-scales related to comfort talking to partner, self-love and sense of future, among a nationally representative sample of 15-24-year olds in the US;^38^ Among low-income girls in the US, having negative experiences related to puberty was linked with lack of information about menstruation, whereas those with positive experiences described learning about menarche as a happy and exciting event^39^	Knowledge about pubertal development, pregnancy, HIV/AIDS, sexual orientation; Knowing where to go for health services; Awareness of sexual and reproductive rights
Gender equitable attitudes	Hold attitudes that support gender-equal norms related to the social and cultural roles, responsibilities, rights and capacities of men and women, boys and girls^1,28,29,40,41^	Attitudes towards gender norms associated with pornography use,^42^ body satisfaction^43^ and mental health^44^ among 10–14-year-olds in multi-site, cross-cultural comparisons	Attitudes and beliefs related to gender norms, roles and relationships; Agreement with sexual double standard; Support for gender equality
Respect for human rights, understanding of consent	Demonstrate respect and empathy for others, understand privacy and consent in relation to self and others^1,13,14,28,29,45,46^	Homophobic teasing in early middle school associated with sexual harassment in later middle school in a US-based sample;^47^ Attitudes supporting wife-beating associated with peer violence victimization among 10-16-year-olds in Armenia.^48^	Attitudes towards sexual minorities and other aspects of sexual and reproductive rights; Attitudes towards sexual consent and sexual violence; Perceived social norms related to sexual identities and preferences.
Critical reflection skills	Ability to critically assess and challenge norms and messages related to gender and sexuality^1,28,29,49–52^	Critical consciousness associated with positive sense of self among a sample of lesbian, gay and bisexual adolescents in Hong Kong;^52^ Critical reflection on cultural, religious and societal values and norms related to sexuality is considered a primary learning objective in international standards for sexuality education^29,50^	Critical consciousness; Critical refection; Critical awareness; Critical action.
Coping and stress management skills	Ability to deal with and learn from negative experiences adversities, handle stress and pressure related to social and sexual expectations^1,28,29,35,53^	Lack of readiness to cope with the onset of menstruation linked with negative puberty experiences among low-income girls in the US;^39^ Skills for mobilising resources (e.g. social, economic, cultural) contributed to reproductive resilience among a sample of 15–19-year-old females in Tanzania^54^	Self-reported coping strategies or skills/resources for dealing with adversities; Having someone to talk about past experiences; Ability to handle stress related to sexuality and development
Interpersonal relationship skills	Ability to communicate, assert values and preferences, and negotiate in both intimate and social relationships^1,9,26,29,46^	Communication about sexual and reproductive health with parents associated with communication with sexual partners and perceived self-efficacy to negotiate safer sex among a sample of adolescent African American females in the US;^55^ Talking with intimate partners, peers or family is linked with different aspects of adolescent sexual wellbeing such as sexual assertiveness, feelings of safety and support^56^	Having talked to someone about SRHR; Knowing how to seek/listen to/respect consent; Understanding how to communicate with others including partners about sexuality


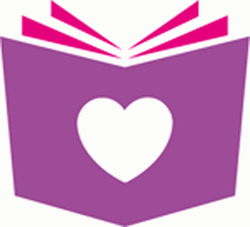
 **Sexual literacy** refers to a developmentally and age-appropriate basic knowledge and access to information related to the human body, development, sexuality and SRHR.^23,28,29^ According to the International Technical Guidance for Sexuality Education, the specific content and meaning of basic understanding will vary according to age and developmental stage; for example, in early adolescence this might include an understanding of the human body and development including puberty, emerging feelings and emotions, the meaning of different relationships (e.g. family, friendships, romantic), and an understanding of gender. Whereas in later adolescence it might expand to knowledge around sexual behaviour, sexual and reproductive health, and where to seek services and support.^29^ The specific SRHR needs and concerns of adolescents vary within and across regions, countries and communities, and this will affect what is considered both appropriate and relevant information.^29^


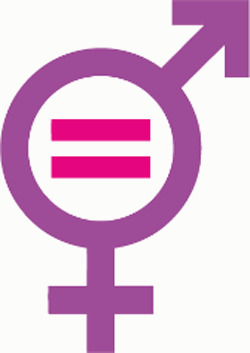
 **Gender equitable attitudes** points to the importance of holding views that support gender equality. As young people transition into adolescence, they are increasingly exposed to social and cultural expectations and messages about the roles, responsibilities, values, power and relationships of men and women, boys and girls. While such gendered norms vary across times and cultures, a set of stereotypical norms prevail in most settings that associate sexual prowess with being a “man” while the same is stigmatised for girls and women.^40,41^ Evidence shows that agreement with such stereotypical norms has consequences for adolescent SRHR, such as masculinity norms pressuring young men to use violence and take sexual risks,^40^ and that perceived gender norms in early adolescence are linked with a host of outcomes related to sexuality and wellbeing such as pornography use,^42^ body satisfaction,^43^ and mental health.^44^ Developing attitudes that support gender-equal norms are thus an important part of healthy sexuality development.


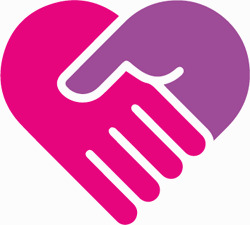
 **Respect for human rights and understanding of consent** highlights the fundamental connection between sexuality and health with human rights,^45^ focusing on the ability to demonstrate respect, tolerance and empathy for others irrespective of their characteristics.^13,14^ Such skills can involve the application of personal values and moral reasoning to distinguish between right and wrong with regard to how to treat ourselves and the people around us. Similarly, as described earlier, applying a rights-based approach to sexuality education has been found to boost positive attitudes towards sexual relationship rights, sexual self-efficacy and communication about sexuality and relationships.^13^ Demonstrating respect for human rights is thus closely connected with ethics, and furthermore about understanding the nature and complexity of consent (e.g. what does it mean to say or give consent?).^1,46^ Evidence indicates that young people are able to define sexual consent and that both boys and girls consider it to be important, but that communicating and interpreting consent signals in real-life situations can be more ambiguous (e.g. considering the absence of verbal refusal as consent).^56^ In the context of our model, respect for human rights might – for example - include positive attitudes towards sexual and reproductive rights, such as the rights of sexual minority groups and to freedom from gender-based violence.


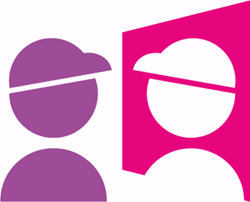
 **Critical reflection skills** can be thought of as the ability to recognise and understand how social norms and structures shape one’s own feelings, behaviours and experiences.^1^ It is not just the cognitive process of reflection, but an understanding of the personal and social world which is shaped via interactions and relationships. Adolescents in most cultural settings are growing up in a context characterised by multiple, and often conflicting, messages and norms related to sexuality and gender – most of them informed by a heteronormative narrative. As a result, adolescents need skills to process different ideas related to what is socially or culturally “ok” (or not) to feel or do (e.g. expecting boys to be sexually active and girls to be abstinent), for whom (e.g. only between men and women), and under what circumstances (e.g. only within marriage).^1,49^ Critical reflection skills are thus essential to allow adolescents to take a step back, reflect and distance themselves from dominant social-sexual norms in way that is in line with their own values.^51^ As noted by Bay-Cheng,^51^ such critical reflection is essential to be able to “plan actions with which to build a world that may better promote their sexuality development and better support sexual thriving”. This involves both the ability to reflect on messages related to one’s own peer group but also to take the perspective of other social groups, e.g. of different gender, ethnicity, social class, sexual orientation.


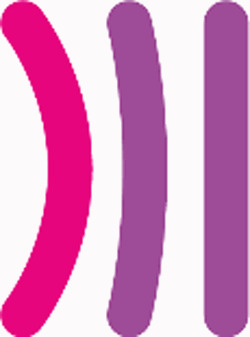
 **Coping and stress management skills** refers to the ability to recognise, manage and adapt to different types of internal or external stressors related to sexuality and development.^53^ Normative adolescent sexuality development involves multiple and often difficult changes,^7^ which – whether or not they are positive or negative – can feel overwhelming and stressful.^1^ Coping is a dynamic process to both manage and adapt to different types of stressful internal or external changes, i.e. it involves skills not only to recognise emotions, but to identity and manage stressors and locate relevant individual or social support.^53^ Coping skills can help adolescents to learn from and translate negative experiences into new ideas and boundaries, as well as seek support in line with their needs,^35^ for example from adults who are close to them, whether a teacher, parent or other professional. Such skills can involve being able to interpret and deal with other people's reactions to one's (sexual) preferences and desires (e.g. stigma against sexual minorities), and to resist and “deconstruct” dominant social and cultural messages related to gender, sexuality and power.^1^


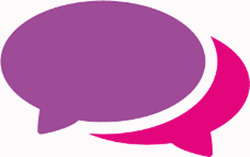
 **Interpersonal relationship skills** include the ability to communicate and negotiate with others, not only in relation to intimate partners, but with peers, family members and others that influence adolescents’ lives. Such skills can include the capacity both to seek information and assert one’s own preferences, as well as being able to listen to and respect others.^1^ Doing so involves the skill to ask for and give consent, as well as refusing and being able to accept refusal of consent.^1,46^ Interpersonal relationship skills may thus help both to prevent unwanted interactions and sexual assault, and to promote positive, pleasurable intimate relationships.^9^ Indeed, a recent review of adolescent sexual wellbeing indicates that young people want skills to talk about SRHR; that sexual communication with partners is associated with (greater) sexual pleasure feelings of safety and support; and that talking with friends and family members about sexuality and relationships has been linked with greater sexual assertiveness and other aspects of sexual wellbeing.^56^

### Sexual wellbeing: personal and relational dimensions

The framework further shows how each of the interconnected healthy sexuality competencies can contribute to adolescent's sense of sexual wellbeing (or lack thereof). Here, we differentiate between *personal* and *relational* aspects of sexual wellbeing and give examples of potential outcomes. Our conceptualisation is in line with the sexual wellbeing domains proposed recently by Mitchell and colleagues,^25^ e.g. comfort with sexuality, sexual self-esteem, and safety and security – albeit with different terminology and adapted specifically for the adolescent population.

*By personal sexual wellbeing*, we mean aspects such as having a positive sense of one’s own (sexual) *self* (e.g. identity, self-esteem, self-efficacy, motivations, feelings and desires) and *body* (e.g. perceived body image, comfort with puberty changes).^1,3,9^ Having a positive sense of self and body is closely linked to the concept of “sexual selfhood” and can include the capacity to be aware of one’s sexual desires,^1,3,7,28^ recognising that it is fine to enjoy these feelings and to experience pleasure,^28^ sexual self-esteem (sense of self-worth and attractiveness) and self-efficacy (perceived ability to assert preferences).^3^ Two central outcomes are self-esteem and self-efficacy related to interpersonal relationships. Self-esteem can be thought of as an individual’s sense of self-worth and attractiveness as well as competence in romantic and sexual situations, whereas self-efficacy refers to the “perceived ability to assert preferences and desires” with a partner, including the desire not to engage in any romantic or sexual activities, and to negotiate condoms and contraceptives to protect against HIV/STIs and unintended pregnancy.^3^ These constructs interact with feelings and experiences related to the development of the physical body including body image and appreciation, level of comfort in relation to body changes that occur in puberty including menstruation.^57,58^

By *relational sexual wellbeing*, we refer to a sense of (sexual) safety and security via mutually respectful relationships that are characterised by gender equality and free from violence and coercion. For adolescents and especially the youngest age groups, having mutually respectful relationships does not only include those with intimate partners, but – and perhaps more importantly – also those with other people who are relevant to their social contexts and who are part of their sexual socialisation, such as parents, peers, relatives and community members.^7^ In the context of this conceptual model, outcomes measuring wellbeing in relation to peers might look at experiences of bullying, teasing, harassment and violence related to gender and sexuality, as well as perpetration of psychosocial and physical types of violence against other peers.^37^ Over time, relational sexual wellbeing can extend into that related to intimate relationships, such as perceived power and communication, self-determination and respect in relation to sexual partners, and resilience related to (past) sexual experiences. ^1,3,6,7,9,28^ As adolescents age and engage in sexual relationships, the links between sexual wellbeing and sexual health (e.g. condom and contraceptive use, sexual function and arousal, freedom from HIV/STIs, prevention of sexual and intimate partner violence violence) and sexual pleasure also become more apparent and relevant from a public health perspective. ^25^

### Sexual agency and navigation


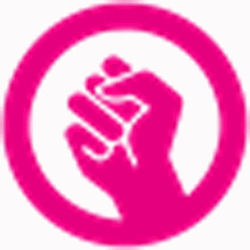
 Whether or not individuals are able to translate competencies into action depends on their *agency* or ability to negotiate which intimate and/or solo sexual activities, if any, to engage in. In the context of this framework, sexual agency forms the link between competencies and wellbeing, and can be thought of as adolescents’ ability to realise preferences and choices, express voice and influence and make decisions by drawing on resources at multiple levels.^30^ We view agency as being relational, with young people’s personal agency being closely shaped by, and shaping, the agency of others in their social contexts.^35^ As described in Arbeit’s skills-based model, sexual agency forms the connection between skills related to self (sexual selfhood) and others (negotiation with intimate partners): thus, personal agency is strongly shaped by, and shapes, the agency of intimate partners. It is also linked with the agency of other people in their social networks (e.g. peers, parents, siblings, relatives, teachers) as well as the broader societal structures and institutions that surround them.

We further recognise that sexual agency is not always positive from the views of an external observer, but that it includes the ability to “endure, suffer and persist”.^35^ Likewise, sexual agency is not something that is equally available to all people – multiple institutional structures restrict individuals’ ability to be their own, autonomous agents, such as laws and policies, social and cultural norms, heteronormativity and inequalities related to race, ethnicity, sex and gender identity.^51^


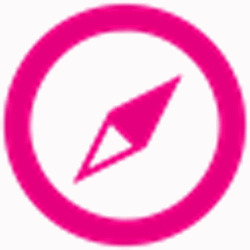
 *Navigation* refers to how young people make decisions about their sexuality in light of multiple goals, circumstances, past experiences, feelings, expectations, sense of benefits and risks, where clear-cut right or wrong answers or decisions are not always apparent or available. While a focus on individuals’ freedom to choose is important from a rights-based perspective, it entails that young people are always in control and responsible, which may not be the case. In other words: making healthy choices and exercising agency related to sexuality can entail making the “wrong” choices, if these are the best available options for the individual at a certain timepoint.^2,35^ For example in a context of poverty and where transactional sex is the norm, adolescents may choose to have unsafe sex because the benefits (of receiving money, gifts, housing, favours, etc) outweigh the risks (of getting pregnant or HIV/STIs) in light of other goals than health, e.g. to bring food back to the family, to get money to start a business, or to strengthen the relationship.

### Social-ecological opportunity structures

Finally, the framework highlights the role of the social-ecological opportunity structures in determining if, how and when adolescents are able to translate competencies into desired actions.^30–33^ While knowledge, skills and attitudes are important, these can be limited or annihilated by an individual’s family, peers, community and broader societal context,^10^ such as having a violent partner, lack of support structures, lack of access to contraception, etc. Opportunity structures exist at multiple, interacting levels including the individual (e.g. experiences, physical health, sociodemographic background), family (e.g. relationships and connectedness with parents, siblings and other relatives), peers (e.g. networks and norms), community (e.g. availability of health services, education, connectedness with teachers) and the broader societal level (e.g. macro factors such as norms, laws, policies and economy).^10^ For example, imagine an adolescent girl who is in love with one of her female peers. Whether or not she is able to translate her knowledge (e.g. about different types of romantic attractions), attitudes (that same-sex relationships are fine) and skills (e.g. to form relationships) to act on her feelings depends on her context, such as: whether homosexuality is legal in her country, whether social norms stigmatise rather than accept same-sex relationships, whether her family would be supportive, or whether she knows peers with similar experiences. These opportunity structures thus play a critical role in determining her agency and sense of sexual wellbeing.

## Conclusion and implications

The importance of investing in adolescent SRHR from a broad perspective was first recognised at the 1994 International Conference on Population and Development (ICPD), which called for “meeting the educational and service needs of adolescents to enable them to deal in a *positive and responsible way with their sexuality*” and highlighted young people’s right to comprehensive sexuality education.^59^ Despite this vision, programmes and research remain largely shaped by a traditional risk approach to adolescent sexuality.^2,4,7,60^ This includes evaluations of comprehensive sexuality education programmes which tend to focus on measuring outcomes related to two main public health indicators (teenage pregnancy and STIs), despite the increased call for measuring “soft outcomes” (such as gender attitudes, relationships skills, etc.) as important in their own right.^13,61^ While the past 25 years have seen significant progress in adolescent SRHR, data and evidence on sexual wellbeing outcomes remain scant – especially in low- and middle-income countries.^60^

In response to the continued push towards positive approaches, this review makes a first attempt at proposing a conceptual framework that can be used to inform both programmes, research and practice. First, the framework can provide concrete direction for designing and evaluating adolescent SRHR initiatives that are rights-based and gender transformative. It can help to articulate a Theory of Change for how CSE programmes, for example, can contribute to sexual wellbeing by strengthening adolescents’ skills to reflect upon and navigate different scenarios, regardless of whether few options are available to them. It shows that merely giving information is not enough, but that adolescents need skills to critically reflect on messages, including those communicated via pornography and media.^62^

Second, the framework can be used to guide the operationalisation of measures that tap into the complex construct of sexual wellbeing from both personal and relational aspects. The Global Early Adolescent Study (GEAS, www.geastudy.org), for example, uses a number of indicators to measure young adolescents’ personal sense of sexual wellbeing (e.g. body image, comfort with pubertal development, self-efficacy), as illustrated in a recent study where we analysed data from the Indonesian arm of the GEAS.^36,63^

Third, our assets-based, life-course approach can further be used to make the case to donors and policy makers for why early investments are needed to achieve wellbeing over time. There is growing attention on considering the value of non-health outcomes in cost evaluations^64^ and the framework can serve as a foundation for moving beyond traditional economic measures such as quality-adjusted life years (QUALYs).

When using the framework, researchers, donors and programme implementers need to consider carefully the broader experiences and context of adolescents’ lives. Young people’s ability to utilise knowledge, skills and attitudes gained via any intervention depends on the resources available to them, their (sexual) agency, and on the influence of opportunity structures. This means that the specific content and framing of many of the domains in the framework will vary depending on the geographical, social, economic and cultural setting where it is being applied, using intersectional approaches and carefully adapting content related to – for example – histories of trauma and marginalisation.^25^ More research is needed to understand sexual wellbeing and what this entails from the perspective of young people themselves according to their social contexts, beyond high-income Western countries where most existing frameworks and data come from today. In light of this, an important next step will be to validate and further refine the framework by engaging with young people, the public and other key stakeholders in different settings globally. Our involvement in the GEAS and partner initiatives such as the Explore4Action programme provides an excellent opportunity to do so, both in relation to empirically testing the framework in relation to a broad range of measures, as well as seeking input from cross-cultural networks of professionals and young people involved in the research and programmes implemented.

Nonetheless, the framework’s grounding in literature on adolescent development as well as internationally agreed definitions of sexuality suggests that it is both relevant and possible to use across different contexts. In some settings it might serve as a starting point for implementers, researchers and policy makers to set priorities and make the case for viewing adolescent sexuality from a positive perspective. In others, it can help to reaffirm and operationalise what medical and public health professionals across the globe have known for decades: that adolescents’ health and wellbeing, including their sexual wellbeing, is more than just the absence of disease.^4^ We hope that the model can serve as a roadmap to realise the ICPD vision of allowing young people to navigate their sexuality in a “positive and responsible” way.^59^
